# Flexible All‐Inorganic Room‐Temperature Chemiresistors Based on Fibrous Ceramic Substrate and Visible‐Light‐Powered Semiconductor Sensing Layer

**DOI:** 10.1002/advs.202102471

**Published:** 2021-10-20

**Authors:** Chaohan Han, Xiaowei Li, Yu Liu, Yujing Tang, Mingzhuang Liu, Xinghua Li, Changlu Shao, Jiangang Ma, Yichun Liu

**Affiliations:** ^1^ Key Laboratory of UV‐Emitting Materials and Technology of Ministry of Education Northeast Normal University 5268 Renmin Street Changchun 130024 China

**Keywords:** flexible substrate, inorganic semiconductors, room temperature, visible‐light‐powered gas sensors, yttria‐stabilized zirconia

## Abstract

As the most extensively used gas‐sensing devices, inorganic semiconductor chemiresistors are facing great challenges in realizing mechanical flexibility and room‐temperature gas detection for developing next‐generation wearable sensing devices. Herein, for the first time, flexible all‐inorganic yttria‐stabilized zirconia (YSZ)/In_2_O_3_/graphitic carbon nitride (g‐C_3_N_4_) (ZIC) gas sensor is designed by employing YSZ nanofibers as substrate, and ultrathin In_2_O_3_/g‐C_3_N_4_ heterostructures as active sensing layer. The YSZ substrate possesses small nanofiber diameter (310 nm), ultrafine grain size (23.9 nm), and abundant dangling bonds, endowing it with striking mechanical flexibility and strong adhesion with In_2_O_3_/g‐C_3_N_4_ sensing layer. Meanwhile, the ultrathin thickness (≈7 nm) of In_2_O_3_/g‐C_3_N_4_ ensures that the inorganic sensing layer has tiny linear strain along with the deformation of flexible YSZ substrate, thereby enabling unusual bending capacity. To address the operating temperature issue, the gas sensor is operated by using a visible‐light‐powered strategy. Under visible‐light illumination, the flexible ZIC sensor exhibits a perfectly reversible response/recovery dynamic process and ultralow detection limit of 50 ppb to toxic nitrogen dioxide at room temperature. This work not only provides an insight into the mechanical flexibility of inorganic materials, but also offers a valuable reference for developing other flexible inorganic‐semiconductor‐based room‐temperature gas sensors.

## Graphical abstract

A unique flexible all‐inorganic yttria‐stabilized zirconia (YSZ)/In_2_O_3_/graphitic carbon nitride (g‐C_3_N_4_) gas sensor is developed by employing YSZ nanofibers as substrate, and ultrathin In_2_O_3_/g‐C_3_N_4_ heterostructures as active sensing layer. Under visible‐light illumination, the flexible sensor exhibits perfectly reversible response/recovery dynamic process to toxic NO_2_ under extreme bending state at room temperature.

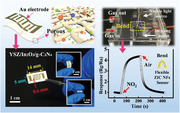

## 1 Introduction

Inorganic‐semiconductor‐based gas sensors, combining with several advantages such as excellent performance, compact size, and easy integration with matured semiconductor technology, have dominated the gas‐sensing field for a long time.^[^ ^1^, ^2^ ^]^ With the thriving development of technologies in portable environment monitoring, wearable expiration diagnosis, and soft electronic nose, developing wearable semiconductor‐based gas sensors with both excellent mechanical flexibility and outstanding sensing performance is becoming an urgent priority.^[^ ^3^, ^4^ ^]^ Currently, to enable mechanical flexibility of semiconductor‐based gas sensors, many conventional flexible substrates including polymers, gels, cellulose nanofibers, were employed to accommodate mechanical deformation.^[^ ^5^ ^]^ However, the weak bond (hydrogen bond or van der Waals interaction) between conventional organic substrates and inorganic semiconductors usually results in undesirable peeling of semiconductor sensing layer from the substrate, seriously affecting the reliability of gas sensors.^[^ ^6^, ^7^ ^]^ Even worse, the poor temperature tolerance of organic substrates seriously hinders their combination with semiconductor‐based gas sensors, which generally require high calcination temperature (400–600 °C) to enhance crystallization and reliability.^[^ ^8^, ^9^ ^]^ Therefore, it is highly desirable to explore next‐generation flexible substrates that can be compatible with semiconductor‐based gas sensors.

Yttria‐stabilized zirconia (YSZ), as a typical dielectric ceramic substrate, has been regarded as an ideal substrate for constructing semiconductor‐based gas sensors.^[^ ^10^, ^11^, ^12^ ^]^ One prominent advantage of YSZ ceramic is that the hanging bonds originating from the fracture of surface lattice provide a good opportunity for strong bonding with semiconductor sensing materials.^[^ ^13^ ^]^ Another superiority is that the high heat resistance (≈1300 °C) makes YSZ ceramic substrate exhibit good compatibility with the thermal treatment of semiconductor‐based gas sensors.^[^ ^14^ ^]^ Unfortunately, as is well‐known, the inherent fragile and rigid nature of dielectric ceramic substrates seriously limits their mechanical flexibility. Some studies suggest that the brittleness of ceramic substrates is derived from large grain boundaries and excessive stress accumulation because the grain boundaries can be seen as underlying microcracks and excessive stress accumulation at grain boundary will extend these microcracks, resulting in irreversible brittle fracture.^[^ ^15^ ^]^ Moreover, from the perspective of structural mechanics, the rigidity of ceramic substrates is believed to stem from the thick film structure, since section modulus in bending (*W* = *bh* ^2^/6) is highly related to the thickness (*h*) and width (*w*) of substrates. Based on the above, to achieve the mechanical flexibility of traditional ceramic substrates, the feasible way is to minimize the grain size and simultaneously change the thick film structure. And to our best knowledge, almost no report focuses on changing the rigid and fragile features of ceramic substrates to make them adaptable with the flexibility of inorganic‐semiconductor‐based gas sensors.

Apart from the achievement of mechanical flexibility, high operating temperature is another issue that needs to be addressed for wearable semiconductor‐based gas sensors, because the high temperature could bring high power consumption and undesired safety issue.^[^ ^16^, ^17^, ^18^ ^]^ Encouragingly, it is acknowledged that the light‐powered strategy provides an alternative method to activate the semiconductor sensing materials and enable the semiconductor‐based gas sensors to work at room temperature.^[^ ^19^, ^20^ ^]^ In particular, the visible‐light‐powered strategy attracts substantial attention, because visible light is a friendly light source for wearable flexible devices.^[^ ^21^ ^]^ Recently, visible‐light‐powered gas sensors have been intensively studied on In_2_O_3_, which has a visible‐light‐responsive indirect bandgap of 2.8 eV.^[^ ^22^, ^23^ ^]^ Although much progress has been achieved, the high recombination rate of photogenerated carriers and the limited visible‐light absorption of pure In_2_O_3_ seriously restrict their gas‐sensing performance. To address this, the most effective solution is to construct In_2_O_3_‐based heterojunction by grafting proper secondary semiconductor with suitable energy band.^[^ ^24^ ^]^ Graphitic carbon nitride (g‐C_3_N_4_) is a metal‐free layered 2D conjugated polymer with an indirect bandgap of 2.7 eV, and thus has visible‐light absorption capacity.^[^ ^25^, ^26^ ^]^ After forming the heterojunction with In_2_O_3_, the visible‐light absorption can be significantly enhanced and the built‐in electric field at the interface is anticipated to promote efficient charge separation.^[^ ^27^ ^]^ Moreover, the layered structure of g‐C_3_N_4_ could facilitate the charge transfer and reduce undesirable recombination of photogenerated carriers.^[^ ^28^ ^]^ Thus, the combination of In_2_O_3_ with g‐C_3_N_4_ shows great potential in realizing high‐performance visible‐light‐powered gas sensors. Whereas, one remaining issue is that both In_2_O_3_ and g‐C_3_N_4_ are inorganic semiconductors, they still suffer from limited fracture toughness. Therefore, it is still challenging to design a visible‐light‐powered In_2_O_3_/g‐C_3_N_4_ gas sensor that possesses both outstanding room‐temperature sensing performance and good conformability to the flexible substrate.

Herein, for the first time, all‐inorganic flexible visible‐light‐powered In_2_O_3_/g‐C_3_N_4_ gas sensors were constructed on electrospun YSZ substrates by atomic layer deposition (ALD) and subsequent vapor deposition method.^[^ ^29^ ^]^ From what we know about the field of inorganic‐semiconductor‐based gas sensors, the superiority of such sensors is as follows: i) the electrospun YSZ substrates possess ultrafine grain size and nanofiber diameter (nm), which can effectively reduce the grain boundary and section modulus in bending, and hence guarantee the mechanical flexibility of the sensor; ii) the In_2_O_3_/g‐C_3_N_4_ sensing layer with the ultrathin thickness (≈7 nm) allows a smaller linear strain compared to conventional bulk semiconductor crystalline, enabling the In_2_O_3_/g‐C_3_N_4_ sensing layer to sustain relatively large deformation along with flexible YSZ substrates; iii) the gas permeability of the YSZ network as well as visible‐light‐powered strategy endow the sensor with fluent gas diffusion and excellent room‐temperature sensing performance. Overall, the as‐obtained flexible YSZ/In_2_O_3_/g‐C_3_N_4_ (ZIC) gas sensor exhibits great potential in wearable gas‐sensing devices. We believe that the proposed strategy for constructing flexible visible‐light‐powered ZIC sensor can be widely extended to other flexible semiconductor‐based room‐temperature gas sensors.

## 2 Results and Discussion

### 2.1 Microstructure and Mechanical Properties

**Scheme 1:**
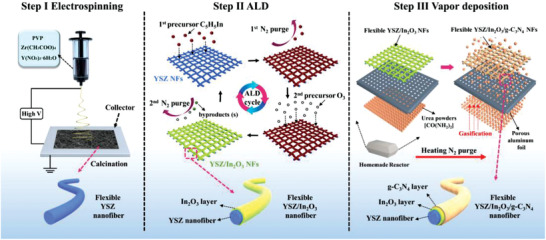
Schematic illustration of the preparation process of flexible YSZ/In_2_O_3_/g‐C_3_N_4_ nanofiber networks. Step I) Electrospinning of the precursor solution and calcination the nanofibers to obtain flexible YSZ nanofibers. Step II) In_2_O_3_ layers were successively deposited on the flexible YSZ nanofibers by using atomic layer deposition (ALD) method. Step III) The g‐C_3_N_4_ layers were deposited on the flexible YSZ/In_2_O_3_ nanofibers by vapor deposition method and the flexible YSZ/In_2_O_3_/g‐C_3_N_4_ nanofiber networks were obtained.

To achieve the flexibility of all‐inorganic semiconductor‐based gas sensors, the YSZ/In_2_O_3_/g‐C_3_N_4_ nanofiber networks was designed by a “bottom to up” strategy, as illustrated in **Scheme** **1**. Initially, the flexible YSZ substrate with good mechanical flexibility was prepared by a facile electrospinning approach. Herein, the ultrafine grain size and small fiber diameter together guaranteed the mechanical flexibility of YSZ substrate, as discussed in detail below. Subsequently, the In_2_O_3_ active sensing layer was deposited in situ on YSZ substrate by ALD. The conformal deposition by ALD technique allows the In_2_O_3_ being deposited on the substrate in an Ångstrom scale and has the thickness of In_2_O_3_ sensing layer under precise control.^[^ ^30^ ^]^ Then, the epitaxial growth of g‐C_3_N_4_ on the surface of In_2_O_3_ was conducted by vapor deposition method and the YSZ/In_2_O_3_/g‐C_3_N_4_ network was finally formed. It is worth noting that instead of traditional mechanical coating of sensing layer on the substrate, the sensing layer was in situ grown on flexible YSZ substrate, which aims to enhance the chemical bonding between substrate and sensing materials, prohibiting undesirable exfoliation of sensing materials from the substrate during deformation.

**Figure 1:**
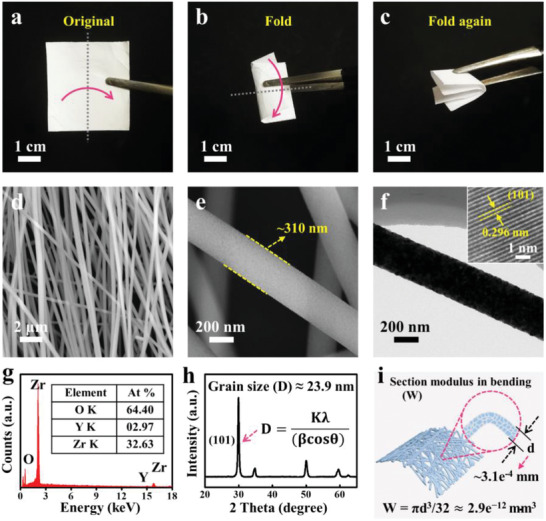
a–c) Digital photographs of the as‐prepared flexible YSZ nanofibers in original, folded, and double folded states. d,e) SEM images of the flexible YSZ nanofibers, and the diameter of the single YSZ nanofiber is about 310 nm. f) TEM image of the flexible YSZ nanofibers, and the inset is the HRTEM image of the flexible YSZ nanofiber, the lattice distance is 0.296 nm. g) Energy‐dispersive X‐ray (EDX) spectrum of the flexible YSZ nanofibers. h) X‐ray diffraction (XRD) pattern of the flexible YSZ nanofibers and the grain size (*D*) of the nanofibers is about 23.9 nm, which is calculated by the Scherer formula (*K* = 0.89, *λ* = 0.154 nm, *β* = 0.38, cos*θ* = 0.866). i) Schematic diagram of the YSZ nanofibers and the section modulus in bending (*W*) is about 2.9*e* ^−12^ mm^3^.

**Figure** **1**a–c presents a piece of the as‐obtained YSZ substrate in folded state. Clearly, no brittle fracture is observed on the ceramic YSZ substrate during such large degree bending, indicating the unusual mechanical flexibility and toughness of YSZ ceramic substrate. Undoubtedly, the abnormal flexibility and toughness of the YSZ substrate are highly related to its microstructure and composition. Accordingly, scanning electron microscopy (SEM) was first conducted and the SEM images indicate that the YSZ substrate is woven by many nanofibers and the nanofibers are charactered by uniform diameter of about 310 nm (Figure 1d,e). The rough surface verified by transmission electron microscopy (TEM) image manifests that YSZ nanofibers are assembled by many tiny well‐crystallized grains (Figure 1f and the inset). Moreover, the energy‐dispersive X‐ray spectrum and X‐ray diffraction (XRD) pattern reveal that the YSZ substrate is composed of Y, Zr, and O elements and can be assigned to tetragonal phase (Figure 1g,h). The calculated average grain size from the half‐peak width of (101) peak by the Scherrer equation (*D* = *Kλ*/(*β*cos*θ*)) was 23.9 nm for YSZ nanofibers. Based on the aforementioned discussion about the rigid nature of conventional YSZ substrate, the ultrafine grain size observed here, on the contrary, can minimize the size of grain boundary as well as the stress accumulation at the grain boundary, which effectively suppress the extension of underlying microcracks (grain boundary) and help to enhance the toughness of the YSZ nanofibers to a certain extent.^[^ ^31^ ^]^ Simultaneously, from the perspective of structural mechanics, the relation between section modulus in bending and diameter of a single nanofiber can be expressed as *W* = *πd* ^3^/32 ≈ 2.9*e* ^−12^ mm^3^, that is, the nanoscaled diameter of YSZ fibers makes the YSZ network extremely soft (Figure 1i). Therefore, it is believed that the ultrafine grain size and diameter of nanofibers together endow the YSZ nanofibers with good toughness and flexibility.

**Figure 2:**
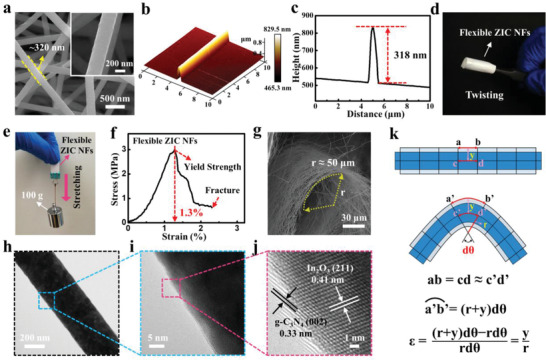
a) SEM images of the ZIC nanofibers (ZIC NFs) and the diameter of the ZIC nanofiber is about 320 nm. b) Atomic force microscopy (AFM) images of the single ZIC nanofiber, c) height profile corresponds to the single ZIC nanofiber in the AFM image, and the height of the ZIC nanofiber is about 318 nm. d) Digital photograph of the flexible ZIC NFs in bending state. e) Photograph of the weights lifted by the ZIC NFs. f) Stress–strain curves of the ZIC NFs. g) SEM image of bent ZIC NFs and the bending radius (*r*) is ≈50 μm. h–j) TEM and HRTEM images of the ZIC nanofiber. k) Schematic diagram of the strain of the flexible nanofiber before and after bending, and the tiny linear strain (*ε*) calculation formula.

Figures S1 and S2 (Supporting Information) present the SEM, TEM, and elemental mapping results of YSZ/In_2_O_3_ (ZI) nanofibers. With the assistance of ALD deposition, the In_2_O_3_ sensing layer was uniformly wrapped on freestanding YSZ substrate. Considering that ALD is a self‐limiting growth technology,^[^ ^30^ ^]^ the successful growth of In_2_O_3_ observed here implies the existence of strong chemisorption between the YSZ substrate and reaction precursors, which is conducive to enhance the adhesiveness of sensing films. **Figure** **2**a,b shows the SEM and atomic force microscopy images of ZIC nanofibers (ZIC NFs). Compared to the pristine YSZ nanofibers (Figure 1e), the ZIC NFs strictly inherited the fibrous microstructure of YSZ substrate, whereas the average diameter of assembled nanofibers increases slightly (Figure 2a,c). The elemental distribution of ZIC NFs manifests that In, O, C, and N elements are evenly distributed on the nanofiber (Figure S3, Supporting Information), demonstrating the uniformly epitaxial growth of In_2_O_3_ and g‐C_3_N_4_ sensing materials. Besides, the photographs about the bending and load bearing indicate that ZIC network possesses excellent bending capacity and mechanical strength (Figure 2d,e). The stress–strain curve manifests that the as‐obtained ZIC networks can sustain a maximum strain level of ≈1.3% (Figure 2f), which is consistent with the previous report about inorganic metal oxide.^[^ ^32^ ^]^ In this context, it is worth mentioning that no significant fracture occurred on the ZIC NFs under the ultimate bending state (bending with the radius of curvature of ≈50 µm), as shown in Figure 2g. And the electrical test indicates that the ZIC network remains stable electric connection under dynamic bending process (Figure S4 and Video S1, Supporting Information), which confirms that the sensing layer has no obvious fracture during large bending state either. To understand this excellent structural toughness, the TEM and high‐resolution transmission electron microscopy (HRTEM) characterizations about ZIC were carried out (Figure 2h–j). The conformal coverage of the In_2_O_3_/g‐C_3_N_4_ sensing layer on YSZ nanofibers was further verified and the thickness of the outermost g‐C_3_N_4_ layer was measured about 2 nm. Moreover, the thickness of In_2_O_3_ sensing layers on YSZ substrate was calculated about 5 nm (0.0125 nm × 400 layers) based on the thickness of the In_2_O_3_ layer that deposited on carbon nanofiber substrate (Figure S5, Supporting Information). It is believed that such ultrathin sensing layers assure the In_2_O_3_/g‐C_3_N_4_ sensing layer of tiny linear strain (*ε*) along with the deformation of flexible YSZ nanofibers. Assuming that the ZIC NFs were under the extreme bending state, as shown in Figure 2g, the radius of curvature (*r*) of the ZIC nanofibers were estimated about 50 µm. The arc length of *a'b’* can be expressed as (*r* + *y*)d*θ*, where *y* represents the radius of ZIC nanofiber, *θ* represents the radius angle of the bending nanofiber. Thus, the elongation of arc *a'b’* (∆*l*) is equal to *y*d*θ* (∆*l* = (*r* + *y*)d*θ* − *r*d*θ*). Correspondingly, the linear strain can be expressed as *y*/*r* (*ε* = [(*r* + *y*)d*θ* − *r*d*θ*)]/*r*d*θ* = *y*/*r*) and the value of *ε* is calculated to be 0.3% (Figure 2k), which is far much less than the maximum strain level of ≈1.3%. Therefore, the ZIC nanofiber can sustain large mechanical deformation and simultaneously maintain a stable electric connection.

### 2.2 Verification of Chemical Composition and Interfacial Interaction

**Figure 3:**
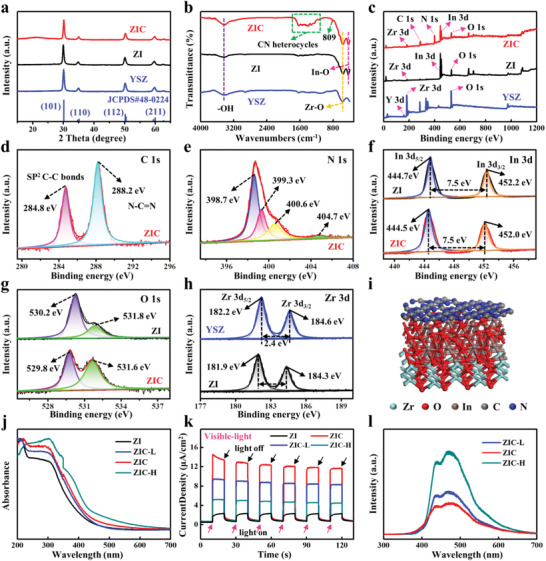
a) XRD patterns of the YSZ, ZI, and ZIC NFs. b) FTIR spectra of YSZ, ZI, and ZIC NFs. c) X‐ray photoelectron spectra (XPS) of YSZ, ZI, and ZIC NFs. XPS spectra of d) C 1s, e) N 1s for ZIC NFs. f) XPS spectra of In 3d, g) O 1s for ZI and ZIC, respectively. h) XPS spectra of Zr 3d for YSZ and ZI, respectively. i) Schematic diagram of chemical interactions between different materials. j) UV–visible diffuse reflectance spectra of ZI, ZIC‐L, ZIC, and ZIC‐H nanofibers. k) Photocurrent responses curves of ZI, ZIC‐L, ZIC, and ZIC‐H nanofibers. l) PL spectra of ZIC‐L, ZIC, and ZIC‐H nanofiber samples under visible‐light illumination.

Besides the material microstructure and mechanical properties discussed above, the detailed chemical composition and interfacial interaction of the ZIC network were further studied. In **Figure** **3**a, the XRD results exhibit a tetragonal crystal structure of YSZ for all samples, and there are no obvious characteristic peaks of In_2_O_3_ and g‐C_3_N_4_. This might be due to the excellent crystallinity of the YSZ substrates and the small proportion of the In_2_O_3_/g‐C_3_N_4_ sensing layer in the ZIC composites. Fourier transform infrared (FTIR) spectrum of ZIC exhibits four characteristic absorption peaks at 1245, 1321, 1413, and 1635 cm^−1^, which were generally assigned to the characteristic stretching modes of the C—N heterocycles (Figure 3b).^[^ ^33^ ^]^ Moreover, the typical peak at 809 cm^−1^ was believed to be assigned to triazine units.^[^ ^34^ ^]^ All of these characteristic peaks verified the formation of g‐C_3_N_4_. Besides, the absorption peaks at 435 nm^−1^ were observed in ZI and ZIC, which was generally ascribed to the presence of In—O phonon vibration.^[^ ^35^ ^]^ And for all of the as‐obtained samples, the peaks at 565 cm^−1^ are ascribed to the typical absorption peaks related to the Zr—O stretching vibrations. To further investigate the chemical structure of the as‐obtained samples, the sensing materials were investigated by X‐ray photoelectron spectroscopy (XPS). As can be seen in Figure 3c, the survey spectra indicate that the C, N, In, O, and Zr elements clearly exist in the ZIC NFs. Because of the facts that the XPS measurement is a typical surface analysis method and that the sensing layer (In_2_O_3_/g‐C_3_N_4_) is covered on the surface of the YSZ nanofibers, the XPS signal of Y and Zr that comes from the internal YSZ nanofiber substrate is weakened. And because the content of the Y element (as dopant) is very small, its XPS peak corresponding to the Y element cannot be identified clearly here. As shown in Figure 3d, the high‐resolution C 1s spectrum exhibits two peaks at binding energies of 284.8 and 288.2 eV, which can be assigned to C—C bonds and N—C═N from the *s*‐triazine rings of g‐C_3_N_4_, respectively.^[^ ^36^ ^]^ Moreover, the four characteristic peaks at 404.7, 400.6, 399.3, 398.7 eV in high‐resolution N 1s spectrum (Figure 3e) are corresponding to the terminal amino groups (—NH_2_), C—NH*_x_*, tertiary nitrogen N—[C]_3_ groups, and C═N—C from g‐C_3_N_4_, respectively.^[^ ^37^ ^]^ These observations further verify the formation of the g‐C_3_N_4_ sensing layer. For the In 3d spectra (Figure 3f), the spectrum of ZI presents two peaks at 444.7 and 452.2 eV, which agrees well with the previous report,^[^ ^24^ ^]^ indicating the existence of In^3+^. Combining the evidence of FTIR spectra as well as the observed lattice distance in Figure 2j, the formation of In_2_O_3_ was also further verified. In addition, after the epitaxial deposition of g‐C_3_N_4_, the In 3d peaks of ZIC NFs shifted to the lower binding energy with the value of about 0.2 eV. Meanwhile, the O 1s peaks of ZIC were also slightly shifted to lower binding energy compared with the ZI nanofibers (Figure 3g). The observed peak shifts of In 3d and O 1s are due to the interface electrical‐field‐modulated electron transfer from g‐C_3_N_4_ to In_2_O_3_ and/or the formation of C—O—In structure during high‐temperature vapor deposition, confirming the strong interactions between the g‐C_3_N_4_ and In_2_O_3_.^[^ ^37^ ^]^ For the Zr 3d spectra (Figure 3h), the peaks centered at 182.2 and 184.6 eV are assigned to Zr 3d_5/2_ and Zr 3d_3/2_ according to previous reports.^[^ ^38^ ^]^ Noteworthy, compared with the bare YSZ nanofibers, the positions of the Zr 3d peaks of ZI also slightly shifted to lower binding energy, suggesting that the In—O—Zr chemical interactions were formed at the interface.^[^ ^39^, ^40^ ^]^ As mentioned above, these chemical interactions at the interface (schematically shown in Figure 3i) will contribute the strong adherence between different materials, which enables the ZIC networks to remain stable even under harsh deformation.

### 2.3 Light Absorption and Charge Separations of In_2_O_3_/g‐C_3_N_4_ Sensing Layer

For light‐powered room temperature gas sensor, the light absorption and photogenerated charge separation are two main processes in affecting the gas‐sensing performance. To figure out how the In_2_O_3_/g‐C_3_N_4_ sensing layer promotes the aforementioned two processes, another two YSZ/In_2_O_3_/g‐C_3_N_4_ samples with lower and higher g‐C_3_N_4_ loading content were prepared (denoted as ZIC‐L and ZIC‐H). The successful fabrication of the YSZ/In_2_O_3_/g‐C_3_N_4_ composites was verified by thermogravimetric analysis (TGA), XRD, FTIR, and SEM characterization. The TGA results show that the loading amount of g‐C_3_N_4_ for ZIC‐L, ZIC, and ZIC‐H is about 6.3%, 10.8%, and 14.7% (Figure S6, Supporting Information). The XRD and FTIR results demonstrate that the chemical composition of these YSZ/In_2_O_3_/g‐C_3_N_4_ composites is identical (Figure S7, Supporting Information). And the SEM images indicate that the diameters of YSZ/In_2_O_3_/g‐C_3_N_4_ samples increase slightly as the g‐C_3_N_4_ content increased (Figure S8, Supporting Information). Furthermore, the light absorption capacity of these YSZ/In_2_O_3_/g‐C_3_N_4_ composites was investigated by UV–vis diffuse reflectance spectroscopy analysis (Figure 3j). As can be seen, the absorption edge of ZI network is about 430 nm. After the epitaxial growth of g‐C_3_N_4_, the obtained ZIC‐L, ZIC, and ZIC‐H networks present obvious enhanced visible‐light harvesting capacity as compared to ZI nanofibers and the absorption edges are gradually redshifted. For visible‐light‐powered gas sensor, the widened visible‐light absorption is very conductive to the production of light‐activated charges and enhancement of gas‐sensing performance.

The effectiveness of charge separation was further evaluated by photocurrent and photoluminescence (PL) spectra. The photocurrent versus time (*I*–*t*) response curves were conducted under several cycles of intermittently visible‐light illumination (Figure 3k). In comparison to ZI, the stronger photocurrent intensity of YSZ/In_2_O_3_/g‐C_3_N_4_ networks indicates that the In_2_O_3_/g‐C_3_N_4_ heterostructure offers better charge separation ability, which can guarantee more photogenerated charges in YSZ/In_2_O_3_/g‐C_3_N_4_ to take part in the gas‐sensing reaction. However, the charge separation ability does not increase monotonically as the g‐C_3_N_4_ content increased. This might be due to the limited mean free path of carriers and more recombination of photogenerated carriers in thicker g‐C_3_N_4_ film. Furthermore, the separation of the photogenerated carriers was investigated by PL spectra (Figure 3l). The lower emission peak intensity of ZIC relative to that of ZIC‐L and ZIC‐H networks indicates the weaker recombination of photogenerated carriers in ZIC, which further demonstrates the better charge separation ability of ZIC network. Therefore, the formation of In_2_O_3_/g‐C_3_N_4_ heterostructure can not only broaden visible‐light absorption, but also promote the charge separation with the aid of built‐in electric field. Such behavior is believed to be crucial for surface gas reaction progress upon exposure to visible light.

### 2.4 Visible‐Light‐Powered Gas‐Sensing Characteristics

**Figure 4:**
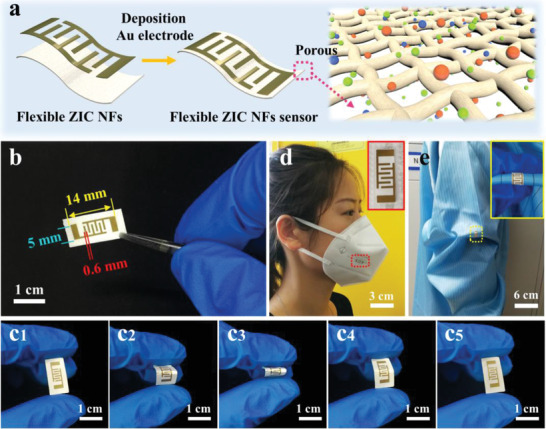
a) Schematic illustration of the flexible ZIC NF sensor and effective NO_2_ gas permeation. b) Photograph of the ZIC NF sensor with Au interdigital electrodes. c1–c5) Exhibition of the flexible ZIC NF sensor. d) Photograph of ZIC NF sensor on the surface of the 3M mask. e) Photograph of ZIC NF sensor on the surface of the experimental clothes with bending.

To evaluate the feasibility of the ZIC network for flexible gas sensor, gold interdigital electrodes with a finger width of 0.6 mm and a gap size of 0.8 mm were directly deposited on the as‐prepared ZIC network and flexible freestanding gas sensors were fabricated (**Figure** **4**a,b). Figure 4c and Video S1 (Supporting Information) clearly show that the flexible gas sensor can sustain very large angle bending and the bending is reversible without fracture. In particular, the fibrous ZIC network endows the flexible freestanding gas sensor with good permeability (Figure 4a and Figure S9 (Supporting Information)), which is very favorable for wearable devices.^[^ ^41^ ^]^ As proof of concept, we attached the ZIC gas sensor on mask and experimental clothes, the conformal deformation along with the mask and clothes clearly shows the practical potential of such flexible gas sensor for future application in portable breathing health diagnosis and wearable environmental sensor (Figure 4d,e).

**Figure 5:**
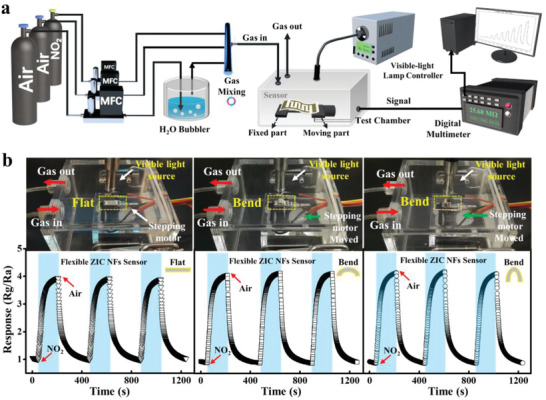
a) Schematic diagram of the homemade dynamic testing platform. The testing platform consists of a stepping motor, including the moving part and fixed part. Fixing the flexible sensor on the stepper motor platform can realize dynamic NO_2_ testing. b) Dynamic gas‐sensing properties of the flexible ZIC sensor for 200 ppb NO_2_ under visible‐light illumination and different bending conditions.

The practical gas‐sensing performances of aforementioned sensors were measured on a homemade dynamic testing platform (schematically illustrated in **Figure** **5**a). The visible lamp with wavelengths ranging from 400 to 700 nm was employed as a visible‐light source. And to control the bending state of the as‐prepared flexible sensors, a motorized translation stage was embedded in the test chamber. The bending state of the gas sensor can be well adjusted by changing the distance between two endpoints of the motorized translation stage. Encouragingly, the flexible visible‐light‐powered ZIC sensor with different bending states maintains the same dynamic sensing performance toward NO_2_ gas (Figure 5b and Figure S10 (Supporting Information)). Furthermore, the flexible sensor can remain in good operating state, even when the sensor is under continuously repeated bending state (Video S2, Supporting Information). These results clearly manifest the potential and feasibility of the ZIC networks as flexible sensing devices powered by visible light.

**Figure 6:**
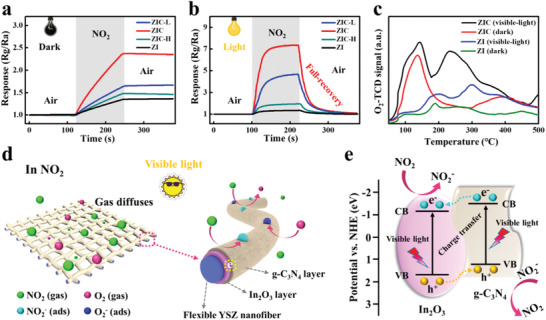
a) The dynamic response curves of the flexible ZI, ZIC‐L, ZIC, ZIC‐H sensors to 1 ppm NO_2_ at room temperature in dark, b) under visible‐light illumination. c) O_2_‐TPD profiles of ZI and ZIC pretreated in dark or under visible‐light illumination. d) Schematic diagrams of the NO_2_ detection process with visible‐light illumination, e) In_2_O_3_/g‐C_3_N_4_ heterojunction and the corresponding energy band diagram.

To further shed light on the promoting effect of visible light and the formation of heterostructure on the gas‐sensing performance, the dynamic NO_2_ responses of the ZI and YSZ/In_2_O_3_/g‐C_3_N_4_ flexible sensors were studied at room temperature with and without light illumination (**Figure** **6**a,b). As can be seen, the sensors based on ZI and YSZ/In_2_O_3_/g‐C_3_N_4_ networks suffer from low response and negligible recovery without light illumination, which seriously hinders their recyclability and practical usage. In stark contrast, the response of sensors increased sharply after exposure to NO_2_ and completely recovered to the initial value after NO_2_ was removed under visible‐light illumination. The response and recovery time was summarized in Table S1 (Supporting Information), it can be observed that the response/recovery dynamic processes are accelerated remarkably under visible‐light illumination. Therefore, the visible‐light illumination can not only help to enhance the gas sensitivity, but also accelerate the response and recovery processes. Compared with the sensor based on ZI, the YSZ/In_2_O_3_/g‐C_3_N_4_ samples process better sensing properties, demonstrating the positive effects of In_2_O_3_/g‐C_3_N_4_ heterostructures in enhancing the visible‐light‐powered sensing performance.

The mechanism about the fast response/recovery speed and the enhanced visible‐light‐powered gas‐sensing response is schematically shown in Figure 6d. The oxygen molecules in air freely diffuse through the porous structure of the ZIC networks and adsorb on the surface of the sensing materials.^[^ ^8^ ^]^ Under visible light illumination, since both In_2_O_3_ and g‐C_3_N_4_ can absorb visible light, a large number of electron–hole pairs will be generated and the adsorption process is promoted, resulting in more adsorbed oxygen molecules.^[^ ^23^ ^]^ Such enhanced oxygen adsorption under light illumination is verified by the O_2_‐ temperature programmed desorption (TPD) results in Figure 6c through comparing the oxygen adsorption with and without light illumination.^[^ ^42^ ^]^ After the gas sensor is exposed to NO_2_, the NO_2_ molecules will capture electrons from the sensing materials directly and/or through the competitive adsorption with the adsorbed oxygen, as described in Equations (1) and (2).^[^ ^43^ ^]^ Since a large number of electrons in the composite are consumed during the gas‐sensing reaction, the number of carriers in sensing layers decreases and the resistance increases rapidly until it reaches a stable value. When the reaction chamber is refilled with pure air, photogenerated holes are beneficial to assist the residual NO_2_ species desorption from the surface of composite materials, as indicated in Equation (3). On the other hand, photogenerated electrons react quickly with physically adsorbed oxygen molecules on the surface to form new chemically adsorbed oxygen. Both these two processes would help the sensor to establish a new dynamic balance. And because of the participation of photogenerated electron–hole pairs in the response/recovery process, the faster response/recovery speed of the sensor can be therefore obtained

(1) $$NO_{2}\left(g\right)\;+\;e^{-}\left(hv\right)\;↔{\text{NO}}_{2}^{-}\left(\mathrm{ads}\right)$$

(2) $$NO_{2}\left(g\right)\;+O_{2}^{-}\left(\mathrm{ads}\right)\;↔{\text{NO}}_{2}^{-}\left(\mathrm{ads}\right)+\;O_{2}\left(g\right)$$

(3) $${\mathrm{NO}}_{2}^{-}\left(\mathrm{ads}\right)\;+\;h^{+}\left(hv\right)\;↔NO_{2}\left(g\right)$$

On the other hand, the enhanced sensing response of the ZIC NFs is believed to be highly related to the improved electron–hole separation by ordered built‐in electric field under visible‐light illumination. As demonstrated by XPS valence band spectra (Figure S11, Supporting Information) and previous reports in the literature,^[^ ^44^ ^]^ the heterostructure between In_2_O_3_ and g‐C_3_N_4_ is a typical II‐type heterostructure (Figure 6e), which means that the photoexcited electrons on the conduction band of the g‐C_3_N_4_ could easily transfer to the conduction band of the In_2_O_3_, similar to the excited holes on the valence band of In_2_O_3_ transferring to that of the g‐C_3_N_4_. Therefore, the photogenerated electrons and holes could be effectively separated, as demonstrated in Figure 3k,l, eventually contributing more carriers to participate in the surface gas‐sensing process and thereby enhancing the sensing response.

**Figure 7:**
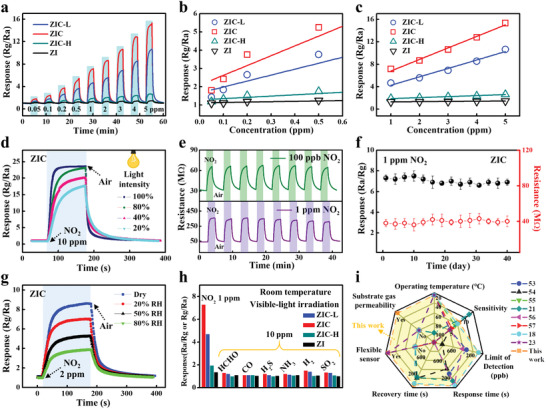
a) Dynamic sensing response curves of ZI, ZIC‐L, ZIC, and ZIC‐H to different concentrations of NO_2_ (50 ppb to 5 ppm) under visible‐light illumination. b,c) The response was fitted with the change of NO_2_ concentration. d) The response of ZIC NF sensor to 100 ppm NO_2_ measured under different visible‐light intensities. e) The repeatability of the ZIC NF sensor toward 100 ppb and 1 ppm NO_2_ at room temperature under visible‐light illumination. f) Long‐term stability of gas sensor for about 40 days. g) The response of ZIC NF sensor to 1 ppm NO_2_ measured under different humidities. h) The selectivity of sensors upon exposure to 10 ppm of various interfering gases. i) The figure of merit for the ZIC NF sensor compared to previous reported NO_2_ gas sensors.

The detailed sensing performance, including the limit of detection (LOD), stability, humidity resistance, and selectivity was further investigated at room temperature with the assistance of visible‐light illumination (**Figure** **7**). The sensor based on flexible ZIC network exhibits significantly enhanced response compared with other sensors (Figure 7a), and the response of ZIC sensor increases more rapidly with the growth of NO_2_ concentration, which makes it prone to distinguish NO_2_ gas with small concentration difference. The LOD can be as low as 50 ppb when the response value greater than 1.5 was employed as the standard for effective gas sensing. Moreover, as can be seen in Figure 7b,c, the response of sensor based on the ZIC NFs (synthesized by using 0.8 g urea, identified by the red squares) has a linear relationship with the NO_2_ concentration ranging from 0.05 to 0.5 ppm (*y* = 5.37*x* + 2.09 (*R* ^2^ = 0.93)). Meantime, this sensor shows a relative smaller slope feature at high concentration (1–5 ppm) (*y* = 2.05*x* + 4.76 (*R* ^2^ = 0.99)). The two linear areas facilitate the sensor to distinguish different concentrations of NO_2_ with a broad range. Noteworthy, for these obtained YSZ/In_2_O_3_/g‐C_3_N_4_ gas sensors, the order of values of response is consistent with the sequence of charge separation shown in Figure 3k,l. That is to say, the better the charge separation, the higher the sensitivity of the samples will be obtained. As a result, it can be also concluded that it is the charge separation rather than the light absorption that contributes more to the gas‐sensing performance. We have the ZIC sensor measured under different intensities of the visible light (20%, 40%, 80%, and 100%). It can be seen from Figure 7d that the response remains nearly the same at 80% light intensity, then drops monotonously as the visible‐light intensity further decreases. This result implies that the employed light intensity is large enough to generate sufficient carriers for surface gas redox reaction and efficiency of light absorption was concealed. Besides, the response and recovery speed slow down slightly with the decrease of light intensity, which further demonstrates that the number of electron–hole pairs generated through light absorption matters a lot toward the response/recovery processes during gas sensing.

Figure 7e shows dynamic resistance curves of the ZIC NF sensor to 100 ppb and 1 ppm NO_2_. The response/recovery properties can be repeated well, and there is no obvious damping of the response value after alternative exposure to NO_2_ gas and dry air. Furthermore, the ZIC sensor was continuously measured for 40 days (Figure 7f). The results verify that the sensor shows no significant fluctuation in response and resistance, suggesting the excellent long‐term stability of ZIC network. During practical application, the background humidity often changes with the ambient environment. Therefore, the humidity resistance performance of the ZIC gas sensor is further examined under different relative humidity (RH) conditions (20%, 50%, and 80% RH) (Figure 7g). As can be seen, although the response curves gradually decreased with increasing humidity, the sensor remains at obvious response even at 80% RH. Many previous reports demonstrated that the water vapor can strongly adsorb on the active sites at room temperature.^[^ ^45^, ^46^ ^]^ The decrease of the response may be due to the competitive adsorption of water vapor and NO_2_ gas near the surface of the sensing materials. Usually, the inherent humidity‐dependent properties of oxide semiconductor gas sensors can be alleviated by many strategies, including using filtering layers and/or changing the test method,^[^ ^47^, ^48^ ^]^ which is beyond the range of current studies. Except for the stability of sensing performance, selectivity is also an important indicator determining the practical application of gas sensors. Figure 7h displays the sensing response of the sensors to 1 ppm NO_2_ and 10 ppm CO, formaldehyde (HCHO), H_2_S, NH_3_, H_2_, and SO_2_ under visible‐light illumination at room temperature, respectively.Notably, the sensor based on flexible ZIC NFs exhibits better selectivity to NO_2_ than other gas, indicating that the sensor is more suitable for NO_2_ detection. This enhanced response toward NO_2_ gas is due to the strong interactions between polarity NO_2_ and g‐C_3_N_4_ (with polar tri‐*s*‐triazine units),^[^ ^49^ ^]^ which promote the adsorption of NO_2_.^[^ ^50^, ^51^, ^52^ ^]^ Furthermore, the gas‐sensing reaction on the surface of the sensing materials is closely related to the type of adsorption of oxygen (O^−^ or O_2_^−^). O^−^ is dominant in the temperature range of 100–200 °C, while O_2_^−^ can exist below 100 °C or even at room temperature. Since the NO_2_ molecule could react with O_2_^−^, the sensing toward NO_2_ commonly exhibits better selectivity at room temperature.^[^ ^50^ ^]^ Moreover, Figure 7i and Table S2 (Supporting Information) highlight the superiority of the designed ZIC sensor from seven aspects in comparison to some recent room‐temperature NO_2_ gas sensors. It is worth noting that the proposed ZIC sensor shows higher room‐temperature sensitivity, lower LOD, and faster response/recovery speed as compared to the previous sensors that work at room temperature or even hundreds of degrees.^[^ ^18^, ^21^, ^23^, ^53^, ^54^, ^55^, ^56^, ^57^ ^]^ More importantly, the unique advantages in gas permeability and mechanical flexibility make it more valuable for application in skin‐attachable wearable sensors than fragile room‐temperature inorganic semiconductor chemiresistors.

## 3 Conclusion

In summary, for the first time, all‐inorganic ZIC flexible visible‐light‐powered gas sensors with both excellent mechanical flexibility and room‐temperature gas‐sensing performance were developed. The YSZ substrate characterized by ultrafine grain size and diameter can effectively reduce the size of grain boundary and section modulus in bending, and hence exhibited good toughness and mechanical flexibility. The ultrathin thickness of the In_2_O_3_/g‐C_3_N_4_ sensing layer endows them with tiny linear strain (*ε*), while the deformation of flexible YSZ substrate, and thus the In_2_O_3_/g‐C_3_N_4_ sensing layer can conformally bend together with flexible YSZ substrate without layer fracture. In particular, the flexible visible‐light‐powered ZIC gas sensors proposed by us exhibited rapid response/recovery characteristics and low detection limits (50 ppb) toward toxic NO_2_ gas molecules, and it remained at a very stable response even under the ultimate bending state. The good mechanical flexibility combining with the excellent room‐temperature sensing performance enable the promising potential of our sensors for future wearable sensing devices. Moreover, the fabrication of the current flexible visible‐light‐powered ZIC gas sensors offers a widely applicable method for constructing all‐inorganic semiconductor‐based gas sensors with excellent room‐temperature sensing performance.

## 4 Experimental Section

### Preparation of Flexible YSZ, YSZ/In_2_O_3_, and YSZ/In_2_O_3_/g‐C_3_N_4_ Nanofibers

0.2 g of polyvinyl pyrrolidone, 10 g of zirconium acetate, and 1 g of yttrium nitrate hexahydrate were mixed and stirred at room temperature for 6 h to make homogeneous spinning solution. The above solution was transferred to a 10 mL plastic syringe, and electrospinning was performed with an applied voltage of 17 kV and a constant distance of 15 cm. The obtained white precursor fiber membranes were calcined at 800 °C for 2 h, and then YSZ flexible nanofibers were obtained. Second, the In_2_O_3_ layer was deposited on the YSZ flexible nanofibers by ALD method. During the synthesis progress, indium cyclopentadiene (C_5_H_5_In) and ozone (O_3_) were used as precursors. The temperature and pressure in the ALD reactor were maintained at 150 °C and 0.1 Torr, respectively. For each deposition cycle, the pulse length of C_5_H_5_In was set to 0.1 s, and the N_2_ purge was 30 s. Next, the O_3_ pulse length was set to 8 s, and the N_2_ purge was 30 s. The number of ALD cycles was 400. Then, the composite nanofibers were calcined at 500 °C at a heating rate of 5 °C min^−1^ for 2 h to obtain YSZ/In_2_O_3_ crystalline flexible nanofibers. Third, the urea (0.6, 0.8, 1.0 g) was evenly spread on the bottom of the ark, and placed on the urea powder with a porous aluminum foil as a support. The distance between the aluminum foil holder and the urea powder was about 6 mm, and 15 mg of YSZ/In_2_O_3_ flexible nanofiber was laid on the aluminum foil. The assembled ark was sealed with tin foil and transferred to a tube furnace at a heating rate of 5 °C min^−1^ and kept at 550 °C for 2 h. Finally, the YSZ/In_2_O_3_/g‐C_3_N_4_ flexible nanofibers were obtained.

### Fabrication and Measurement of Gas Sensor

The fabrication route of gas sensors was as follows: Au electrodes were deposited using magnetron sputtering (AMOD, Angstrom Engineering Inc.) through a shadow mask of stainless steel. The gap between the interdigital electrodes was about 0.8 mm, and the electrode finger width was 0.6 mm. The sensing properties of gas sensor were measured by the homemade system under laboratory conditions. The test system was a self‐made dynamic gas distribution test system, and used a xenon lamp to illuminate the device. The distance from the device was about 5 cm. The power of the xenon lamp was about 100 W, the wavelength was 400–700 nm, and the light intensity was about 137 mW cm^−2^ at room temperature. Here, the response (*S*) of the sensor was defined as *S* = *R*_a_/*R*_g_ or *S* = *R*_g_/*R*_a_, where *R*_a_ and *R*_g_ represent the resistance value of gas sensors when exposed to air or testing gases. The response time and recovery time were the time needed by the sensor for reaching 90% of resistance variation in the case of adsorption and desorption, respectively.

### Statistical Analysis

The data used for the calculation of responses were not preprocessed before the analysis. Each response point was the average of three calculated responses, and the standard deviation was used for evaluating the reproducibility. Statistics were performed using the software OriginPro 9.0 (OriginLab Corporation).

## Conflict of Interest

The authors declare no conflict of interest.

## Supporting information

Supporting Information

Supplemental Video 1

Supplemental Video 2

## Data Availability

Research data are not shared.
