# Supplementary material for: Flexible All‐Inorganic Room‐Temperature Chemiresistors Based on Fibrous Ceramic Substrate and Visible‐Light‐Powered Semiconductor Sensing Layer
Source: Adv Sci (Weinh). 2021 Oct 20;8(23):2102471. doi: 10.1002/advs.202102471 (PMC8655210; doi:10.1002/advs.202102471)
Supplement: Supplementary file 1 — Supporting Information [file ADVS-8-2102471-s001.pdf]

ADVANCED SCIENCE
Open Access

Supporting Information

for *Adv. Sci.*, DOI: 10.1002/advs.202102471

Flexible all-inorganic room-temperature chemiresistors based on fibrous ceramic substrate and visible-light-powered semiconductor sensing layer

Chaohan Han, Xiaowei Li, Yu Liu, Yujing Tang, Mingzhuang Liu, Xinghua Li, Changlu Shao*, Jiangang Ma and Yichun Liu*

Supporting Information

Flexible all-inorganic room-temperature chemiresistors based on fibrous ceramic substrate and visible-light-powered semiconductor sensing layer

*Chaohan Han, Xiaowei Li**, *Yu Liu, Yujing Tang, Mingzhuang Liu, Xinghua Li, Changlu Shao**, *Jiangang Ma and Yichun Liu*

C. Han, Dr. X. Li, Y. Liu, Y. Tang, M. Liu, Prof. X. Li, Prof. C. Shao, Prof. J. Ma, Prof. Y. Liu
Key Laboratory of UV-Emitting Materials and Technology of Ministry of Education
Northeast Normal University
5268 Renmin Street, Changchun 130024, China.
Email: lixw447@nenu.edu.cn;
Email: clshao@nenu.edu.cn;

Characterization

The morphologies of the as-prepared nanofibers were characterized by scanning electron microscopy (SEM; Quanta 250 FEG XL-30; America) and high-resolution transmission electron microscopy (HRTEM; JEOL JEM-2100; Japan). X-ray diffraction (XRD) patterns of the samples were recorded on a Rigaku, D/max-2500 X-ray diffractometer. X-ray photoelectron spectroscopy (XPS) measurement was performed on a VG-ESCALAB LKII instrument (VG, Waltham, UK) with Mg KαADES (hυ = 1253.6 eV) source at a residual gas pressure of below 10^{-8} Pa. AFM measurements were carried out on a Dimension Icon instrument using a NanoScopeV9 controller (Bruker, Inc.). The mechanical properties of the ZIC NFs were measured with a universal material testing machine (zwickiLine Z1.0 T N, Zwick/Roell). The O_2-TPD (temperature programmed desorption) experiments were performed on an automated chemisorption analyzer (Biaode PCA-1200).

Experimental Video

Video S1. Electrical resistance of the ZIC sensor under continuously repeated bending state.

Video S2. Dynamic visible-light-powered gas sensing response of the flexible ZIC sensor under continuously repeated bending state.

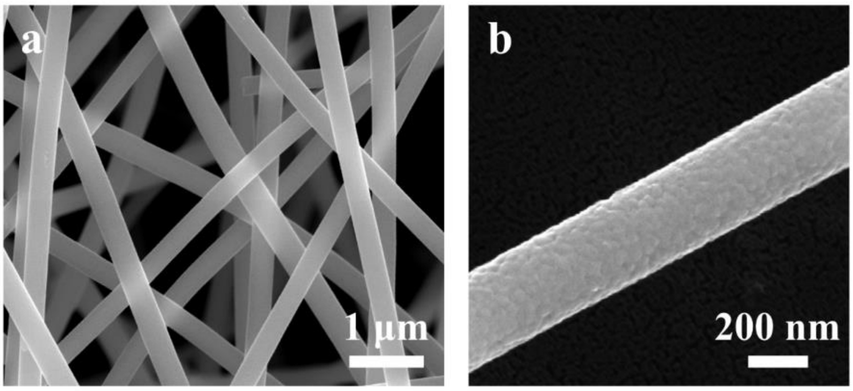

Figure S1 a) SEM image, b) high magnification SEM image of ZI nanofibers.

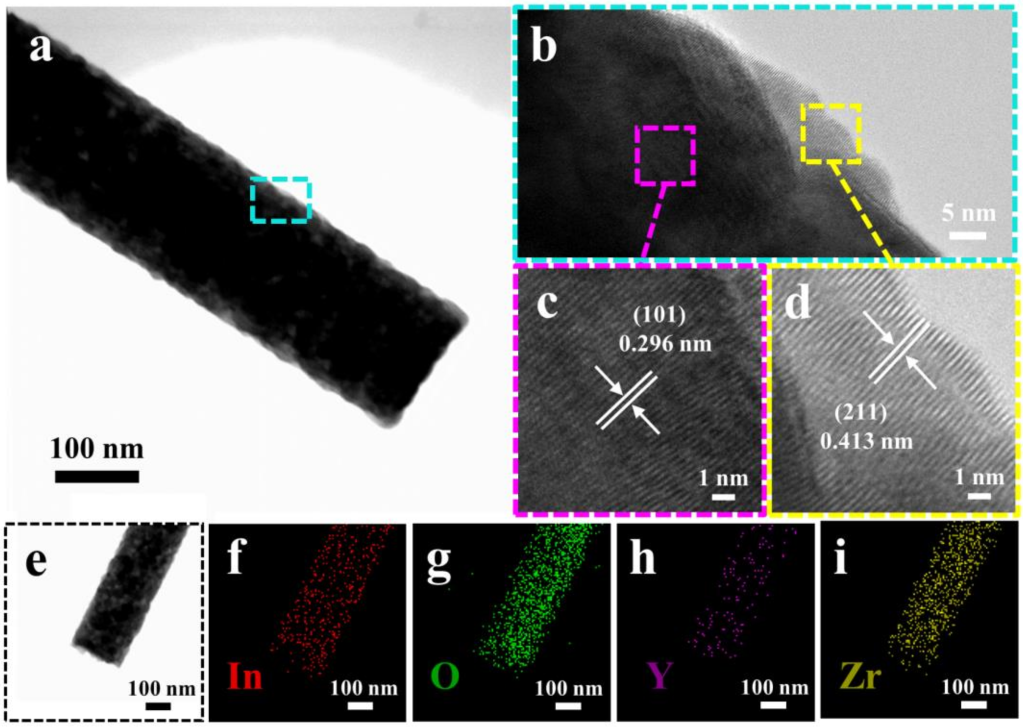

Figure S2 The transmission electron microscopy (TEM) technique was conducted to give a more detailed observation of the YSZ/In_2O_3 nanofibers. a) The In_2O_3 nano-layers were uniformly grown on the surface of the YSZ nanofibers. b-d) The HRTEM presents two different lattice spacing of 0.296 and 0.413 nm, corresponding to the (101) lattice planes of ZrO_2, and (211) lattice planes of monoclinic In_2O_3, respectively. e-i) The elemental mapping images of the YSZ/In_2O_3 nanofibers showed that the In, O, Y and Zr elements were uniformly dispersed on nanofibers.

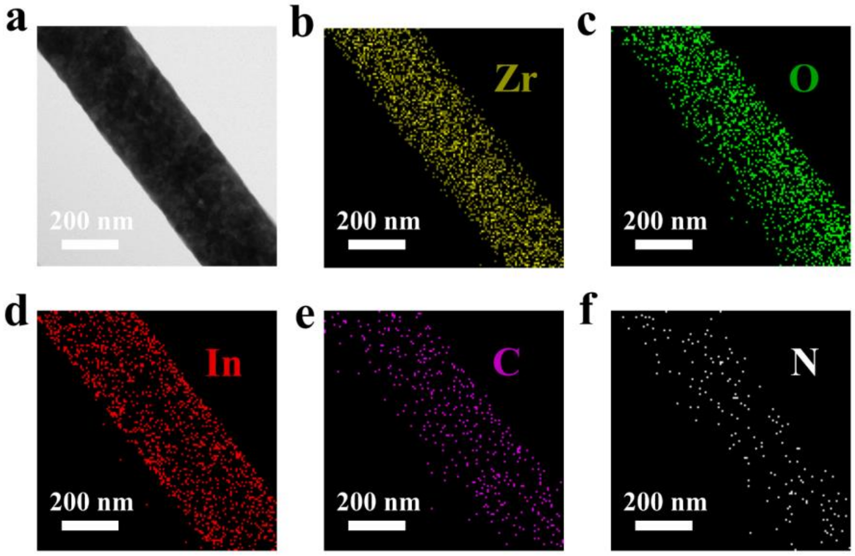

Figure S3 a) TEM image of ZIC nanofiber, b-f) Elemental mapping images of ZIC nanofiber.

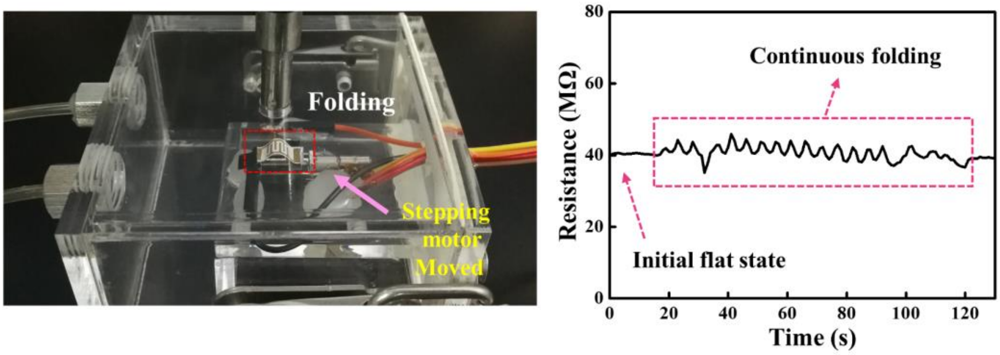

Figure S4 Electrical connection performance test of ZIC sensor under repeated folding.

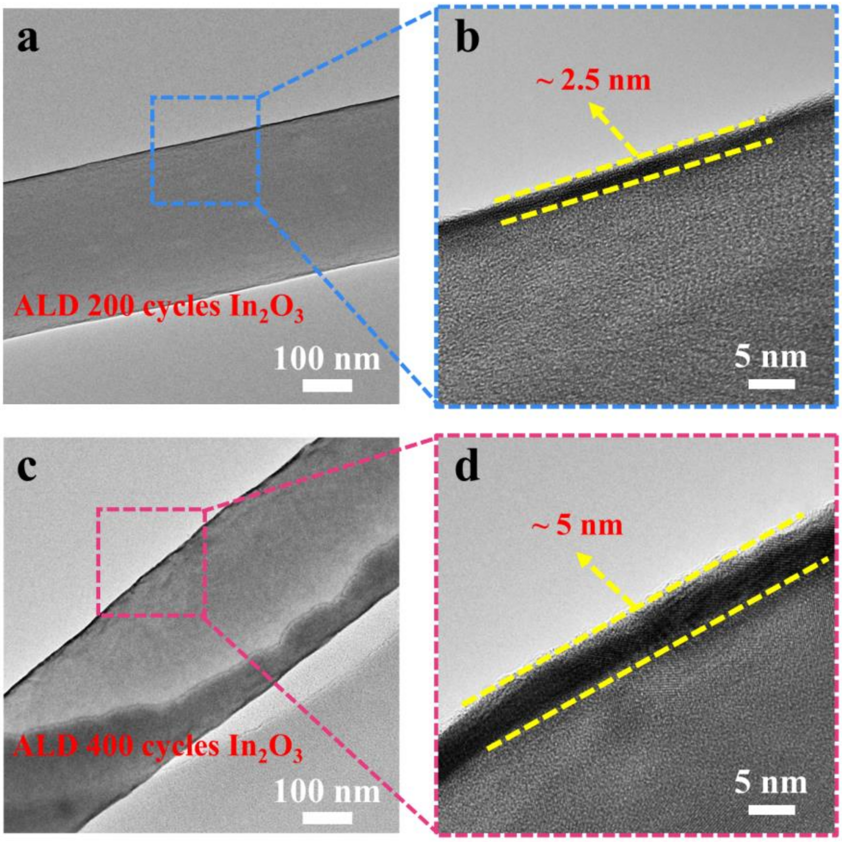

Figure S5 a) TEM images, b) HRTEM of CNFs/In_2O_3 nanofibers with ALD 200 cycles of In_2O_3. c) TEM images, d) HRTEM of CNFs/In_2O_3 nanofibers with ALD 400 cycles of In_2O_3.

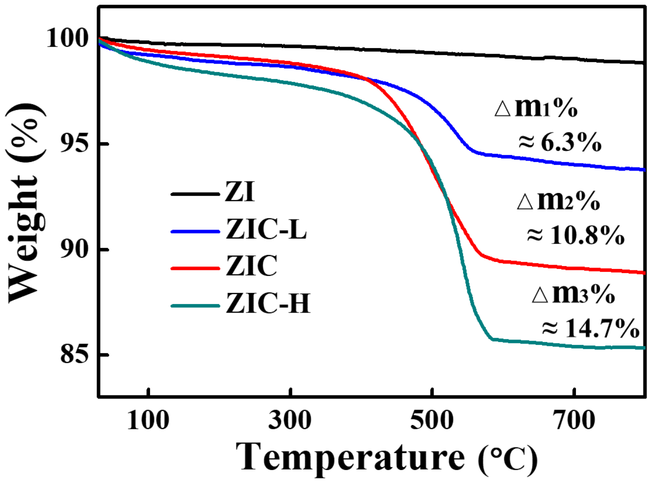

Figure S6 TG curves of ZI, ZIC-L, ZIC, and ZIC-H networks, respectively.

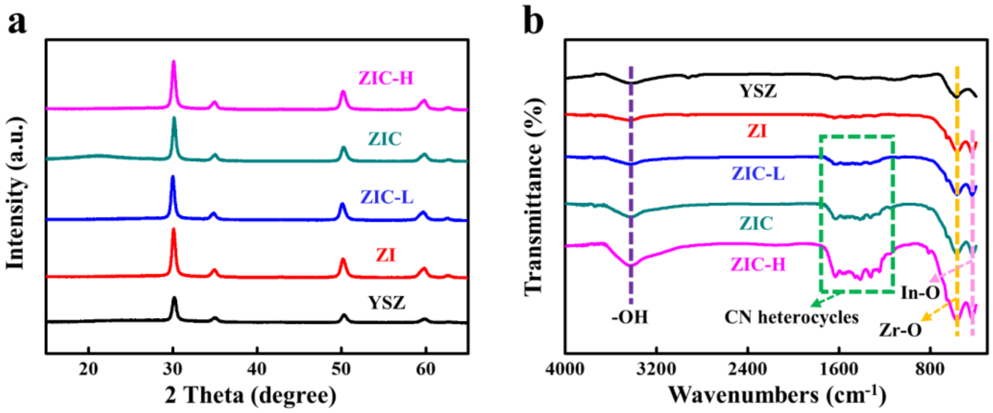

Figure S7 a) XRD patterns, b) FTIR spectra of YSZ, ZI, ZIC-L, ZIC, and ZIC-H networks.

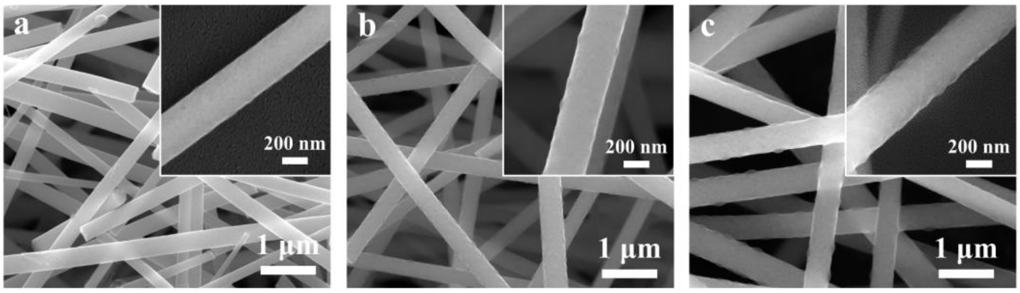

Figure S8 SEM images of a) ZIC-L, b) ZIC, c) ZIC-H networks.

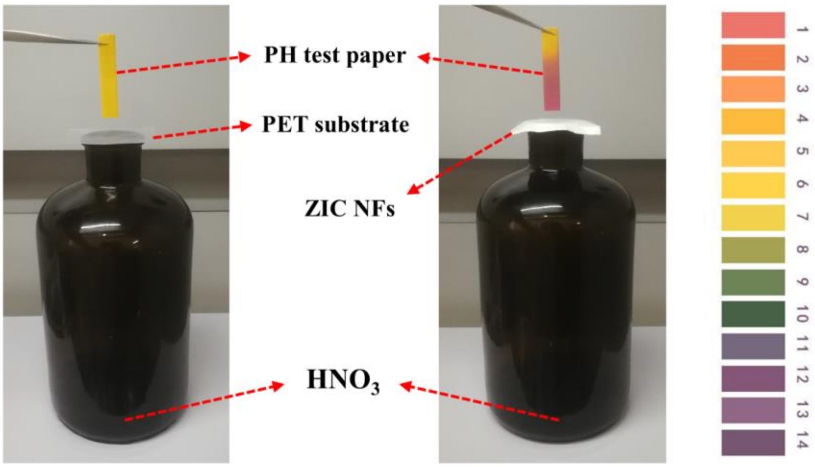

Figure S9 Gas permeability performance test of ZIC networks.

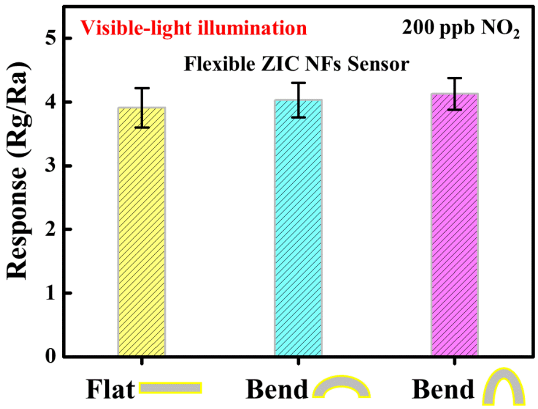

Figure S10 Response of the flexible ZIC sensor to 200 ppb NO_2 at different bending states.

Table S1 The response and recovery time of the as-obtained samples with or without visible-light illumination.

**Sensing materials**	**Response time in dark (s)**	**Recovery Time in dark (s)**	**Response time under visible-light illumination (s)**	**Recovery time under visible-light illumination (s)**
**ZI**	106	-	30	41
**ZIC-L**	110	-	51	67
**ZIC**	113	-	36	80
**ZICV-H**	112	-	32	86

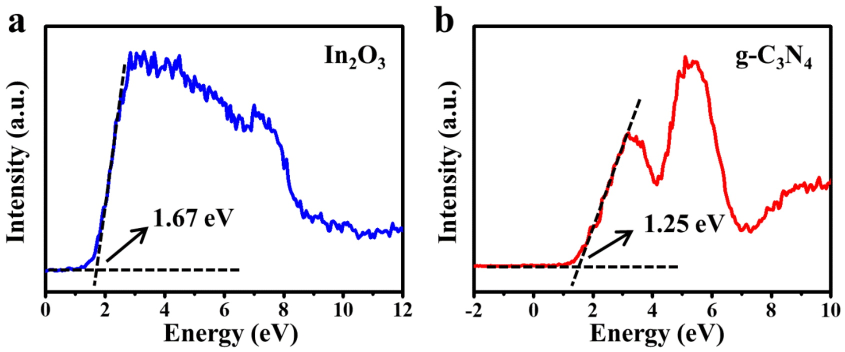

Figure S11 XPS valence band spectra of pure a) In_2O_3, b) g-C_3N_4.

Table S2 Comparison of NO_2 sensing properties of the current work with other reported literatures.

Sensing materials	Operating Temperature (°C)	Concentration (ppm)	Sensitivity (Rg/Ra)/ (Ra/Rg)	Response/ Recovery time (s)	LOD (ppb)	Flexible sensor	Gas permeability	Ref.
**In_2O_3-rGO**	RT	1	1.32	-/-	-	Yes	No	[53]
**WS_2-carbon nanofiber**	RT	1	1.15	>300s/>900 s	200	No	No	[54]
**MoS_2/SnO_2**	RT	10	1.28	408/162	500	No	No	[55]
**ZnO/Au**	RT	1	4.66	-/-	-	No	No	[21]
**Pd-functionalized ZnO nanowires**	100	1	13.5	141/177	-	No	No	[56]
**PPy/N-MWCNT**	RT	5	1.25	65/-	250	Yes	No	[57]
**SnO_2/ZnO nanowires**	RT	1	2.38	-/-	-	No	No	[18]
**In_2O_3/ZnO YS NFs**	RT	1	6.0	36/68	50	No	No	[23]
**YSZ/In_2O_3/g-C_3N_4**	RT	1	7.2	31/44	50	Yes	Yes	This work

References

[18] S. Park, S. An, Y. Mun, C. Lee, *ACS Appl. Mater. Interfaces* **2013**, *5*, 4285.

[21] J. Wang, S. Fan, Y. Xia, C. Yang, S. Komarneni, *J. Hazard. Mater.* **2020**, *381*.

[23] C. Han, X. Li, Y. Liu, X. Li, C. Shao, J. Ri, J. Ma, Y. Liu, *J. Hazard. Mater.* **2021**, *403*, 124093.

[53] R. You, D. D. Han, F. Liu, Y. L. Zhang, G. Lu, *Sensors Actuators, B Chem.* **2018**, *277*, 114.

[54] J. H. Cha, S. J. Choi, S. Yu, I. D. Kim, *J. Mater. Chem. A* **2017**, *5*, 8725.

[55] S. Cui, Z. Wen, X. Huang, J. Chang, J. Chen, *Small* **2015**, *11*, 2305.

[56] X. Chen, Y. Shen, P. Zhou, S. Zhao, X. Zhong, T. Li, C. Han, D. Wei, D. Meng, *Sensors Actuators, B Chem.* **2019**, *280*, 151.

[57] B. Liu, X. Liu, Z. Yuan, Y. Jiang, Y. Su, J. Ma, H. Tai, *Sensors Actuators, B Chem.* **2019**, *295*, 86.
